# Overview of Electrospinning for Tissue Engineering Applications

**DOI:** 10.3390/polym15112418

**Published:** 2023-05-23

**Authors:** Muhammad Zikri Aiman Zulkifli, Darman Nordin, Norazuwana Shaari, Siti Kartom Kamarudin

**Affiliations:** 1 Department of Chemical & Process Engineering, Faculty of Engineering & Build Environment, Universiti Kebangsaan Malaysia, Bangi 43600, Selangor, Malaysia; 2 Full Cell Institute, Universiti Kebangsaan Malaysia, Bangi 43600, Selangor, Malaysia

**Keywords:** tissue engineering, electrospinning, nanofibrous scaffold

## Abstract

Tissue engineering (TE) is an emerging field of study that incorporates the principles of biology, medicine, and engineering for designing biological substitutes to maintain, restore, or improve tissue functions with the goal of avoiding organ transplantation. Amongst the various scaffolding techniques, electrospinning is one of the most widely used techniques to synthesise a nanofibrous scaffold. Electrospinning as a potential tissue engineering scaffolding technique has attracted a great deal of interest and has been widely discussed in many studies. The high surface-to-volume ratio of nanofibres, coupled with their ability to fabricate scaffolds that may mimic extracellular matrices, facilitates cell migration, proliferation, adhesion, and differentiation. These are all very desirable properties for TE applications. However, despite its widespread use and distinct advantages, electrospun scaffolds suffer from two major practical limitations: poor cell penetration and poor load-bearing applications. Furthermore, electrospun scaffolds have low mechanical strength. Several solutions have been offered by various research groups to overcome these limitations. This review provides an overview of the electrospinning techniques used to synthesise nanofibres for TE applications. In addition, we describe current research on nanofibre fabrication and characterisation, including the main limitations of electrospinning and some possible solutions to overcome these limitations.

## 1. Introduction

Tissue repair by transplantation is one of the most promising techniques for tissue regeneration. Transplantation involves the removal of tissue or organ from one person (the donor) and its surgical transplantation into another person (the recipient) or its movement from one site to another site in the same person. There are two types of transplantations, namely autologous and allogeneic. An autologous transplant refers to tissue or organ from the same person who will receive the transplant. It is most often used to treat blood cancers, such as lymphoma and leukaemia. Some drawbacks of autologous methods are limited availability and donor site morbidity. An alternative to autografts is allograft transplants which involve the transplant of tissue or organ from one person to another. Allografts are used to treat and repair damaged limbs, tendons, heart valves, skin, corneas, etc. However, they come with the risk of disease transfer and have the potential to cause an immune response. Therefore, tissue engineering (TE) has emerged as an excellent approach for the regeneration or repair of damaged tissue, with the potential to overcome all the limitations of autologous and allogenic tissue repair [1].

**Figure 1:**
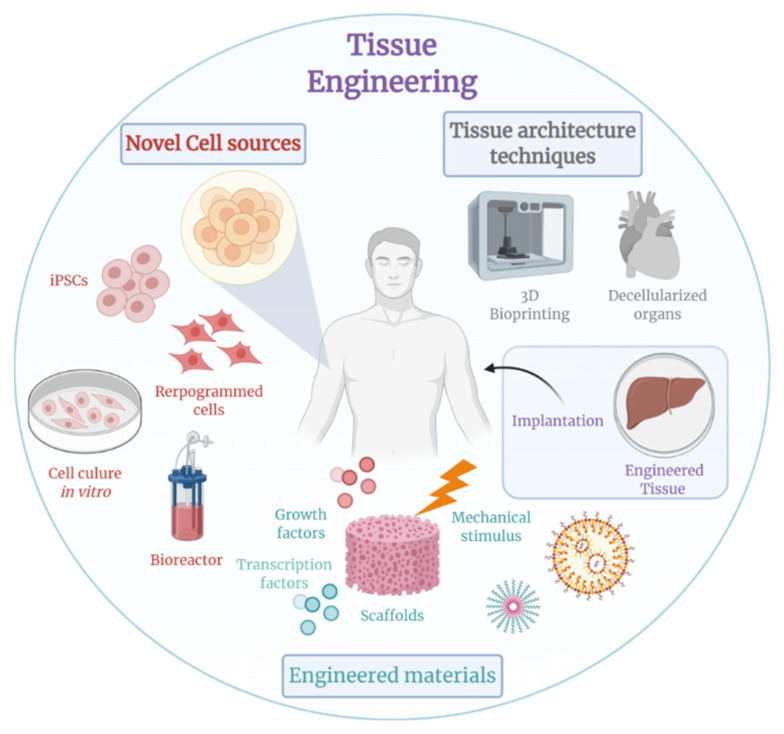
Tissue engineering.

TE represents an emerging interdisciplinary field that applies the principles of engineering and life sciences toward the goal of tissue regeneration [2]. TE approaches make use of cells, biomaterials, or a combination of both to maintain, repair, or improve tissue function, as shown in Figure 1. For example, temperature-responsive polymer brush coatings (TRPBCs) are one of the advanced biomedical applications that use a combination of cells and biomaterial for TE treatment [3].

There are three main approaches that were originally described for tissue engineering and are continually being refined [4]. The first approach is to guide tissue regeneration using only engineering matrices. In this approach, a scaffold is typically implanted at the site of interest, and cells are migrated from the surrounding tissue and implanted into the scaffold. A second approach is then to introduce only allogeneic, autologous, or xenogeneic cells to the site of interest. This approach has the advantage of being minimally surgically invasive, and the cells can be manipulated by clonal propagation or recombinant genetic engineering prior to infusion or injection. Finally, a third approach is to build cells that are placed on or within a matrix. In a third approach, cellular constructs seeded on biodegradable scaffolds intended to act as synthetic extracellular matrices are implanted at repair sites within the body. In fact, organ-specific cells are often seeded into scaffolds ex vivo prior to transplantation, and over time the scaffolds are simultaneously degraded so that the cells synthesise new extracellular matrix and eventually produces new well-functioning tissue [3].

The ability of TE to regenerate the organs and tissue of a patient that are completely free of bad biocompatibility, low bifunctionality, and severe immune rejection is one of its special benefits. Because of these special advantages, TE is often considered an ultimately ideal medical treatment [5]. Biomaterials are important in TE because they act as scaffolds or matrices for cellular proliferation and adhesion. As the primary goal of the production of scaffolds via TE is to mimic a natural extracellular matrix (ECM) at the nanoscale in order to provide a more favourable environment for cell proliferation and attachment, several novel approaches have been developed for the fabrication of biomaterial-based 3D scaffolds.

Presently, electrospinning, self-assembly, and phase separation are the three primary methods accessible for the manufacture of nanofibrous scaffolds using TE. An overview of the benefits and drawbacks of the primary fabrication methods employed to synthesise a nanofibrous scaffold may be seen in Table 1. The approach that has been the subject of the greatest research and appears to provide the best prospects for TE applications is electrospinning. There have been just a few studies that looked at the use of self-assembled and phase-separated nanofibres as TE scaffolds [1]. Since the invention of electrospinning, numerous polymers have been successfully electrospun for a variety of applications due to the technique’s many benefits, including high porosity, a high surface-to-volume ratio, and the simplicity with which the appropriate fibre morphology and mechanical strength may be achieved. Additionally, the electrospinning method itself is flexible since a variety of polymers can be used to spin the fibre into any desired shape [6]. Drug delivery, wound healing, biomedical applications, environmental engineering, food packaging, and TE tissue are just a few areas where electrospun nanofibres are extensively used [7,8,9,10,11,12]. Recently, nanofibre-based scaffolding technologies have come under investigation as TE scaffolds [13,14,15,16,17]. The capacity of the approach to create scaffolds that may be able to replicate the ECM, together with the high surface area to volume ratio of the nanofibres, encourages cell migration, proliferation, adhesion, and differentiation, all of which are highly desired features for TE applications. As a result, the electrospinning method used to create nanofibres for TE applications is the main emphasis of this review. The review also offers highly useful information on the current research on the fabrication and characterisation of nanofibres. The uniqueness of this review paper is that not only it contains updated findings in detail on the application of electrospinning in TE but the major electrospinning limitation for TE applications and several possible solutions to solve these limitations were also included and discussed in detail.

## 2. Electrospinning Technique

Electrospinning is a fabrication technique used to synthesise fibres and is a top choice for researchers as it can be applied to various polymers in addition to having the ability to produce fibres in the submicron range [6]. The dry spinning process was involved in the electrospinning technique, which uses electrostatic forces to pull fibres from a polymer solution [21]. Electrospinning has the advantage of producing fibres with a high surface-to-volume ratio, consistent structure, tuneable porosity, and malleability to conform to a wide range of sizes and forms [22].

### Development of Electrospinning

**Figure 2:**
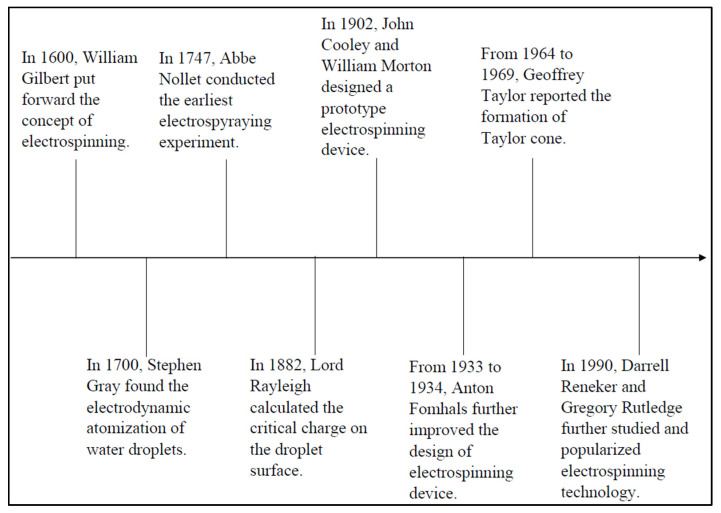
Early history of the electrospinning technique.

Prior to the existence of the electrospinning technique, researchers used the electrospraying technique to disperse solutions and fine aerosols for the synthesis of fibres via electrical energy. Figure 2 below shows the early history of the electrospinning technique [23]. Rayleigh was the first to observe it in 1897, and an in-depth study was conducted by Zeleny in 1914 [6]. One of the earliest patents for electrospinning polymers was issued to Formhals in 1934. Later, Formhals published a series of patents between 1934 and 1944 on experimental approaches for the creation of polymer filaments utilising electrostatic forces [24]. Furthermore, Antonin Formhals patented the production of yarn textiles from cellulose acetate via the electrospinning process. The solvents used in this process are acetone and ethylene glycol monomethyl ether. The concept used by Formhals in the electrospinning process is that a movable yarn-collecting device collects the yarn in a stretched state [25]. Later, the findings of Taylor’s electrical-driven jet research in 1969 led to the rapid development of the electrospinning process [26]. Plenty of research groups published their work on electrospinning in the period 1970–1996, either exploring its possible applications or investigating the process itself [27]. The electrospinning process has become very popular since the 1980s in fibre production because it makes it simple to produce nanofibres composed of various polymers. Currently, more than 200 universities and research institutions around the world are studying various aspects of the electrospinning technique [28].

## 3. Polymers Used in Electrospinning

### 3.1. Natural and Synthetic Polymers

In the last ten years, electrospinning of polymers has been intensively studied as it allows fibres to be synthesised from a variety of polymers such as synthetic polymers, natural polymers, or a mixture of both types of polymers, which is known as a copolymer.

Naturally, polymers normally exhibit low immunogenicity and better biocompatibility compared to synthetic polymers when used in biomedical applications [6]. Typical natural polymers used for electrospinning include gelatin, cellulose, chitin, silk, and wool, which promise better clinical functionality [29,30,31,32,33,34,35]. However, the disadvantages of using nature polymers for electrospinning are partial denaturation and fabrication problem of some natural polymers.

Synthetic polymers have several benefits over natural polymers since they can be designed to provide a wider variety of features, such as desired breakdown rate and required viscoelasticity and strength. Meanwhile, the drawbacks of using synthetic polymers for electrospinning are long biodegradation and low biocompatibility of some synthetic polymers. The typically used synthetic polymers for electrospinning include polyglycolide, polycaprolactone, polylactide, polyurethane, polystyrene, and polyvinyl alcohol.

### 3.2. Copolymers

Copolymers have recently been discovered to be more beneficial than homopolymers because they can be added to change the biodegradation and physical properties of nanofibres [36]. Electrospinning with copolymers offers the improvement of various properties of polymeric materials, including mechanical strength, barrier properties, and the tailoring of thermal stability. Copolymers are used to synthesise a new material having the desirable fibre properties; when the electrospinning technique is properly implemented, the performance of the electrospun scaffolds based on copolymers can be significantly improved as compared to that of homopolymers. Biodegradable hydrophobic polyesters commonly have good mechanical properties but lack the cell affinity for TE. However, with the combination of an appropriate hydrophilic polymer segment, the lack of cell affinity can be solved as hydrophilic polymers enhance cell affinity. Moreover, the morphology, structure, biodegradability, and mechanical properties can also be customised using copolymers in electrospinning [6]. For example, an engineering material with high mechanical strength, poly (methyl methacrylate) (PMMA), has limited use because it decomposes at approximately 250–300 °C in air. However, when the PMAA matrix is incorporated with methacrylic acid, its degradation temperature increases by 80 °C [37]. Furthermore, the presence of sodium copper chlorophyllin (SCC) inside polycaprolactone (PCL) nanofibres provides adequate space for the living cells in the scaffold [22]. The potential of polycaprolactone (PCL)/hydroxyapatite (HA) nanofibres for TE scaffold applications has also been studied. The addition of HA results in bead-less fibres without agglomerates, with the fibres being thicker than the PCL nanofibres. Furthermore, scanning electron microscopy (SEM) images have shown that the scaffolds are biocompatible, suggesting that PCL/HA is a suitable candidate for scaffolds in TE applications [38]. As a result, copolymer-based electrospinning appears to be an appealing option for improving the characteristics of polymers for TE applications.

## 4. Solvent Used for Electrospinning

The dissolving of the polymer in a suitable solvent is the initial step in the electrospinning process. Solvents must have good boiling points, volatility, vapour pressure, and other properties and must maintain the integrity of the polymer solution. This is due to the fact that the solvent that is used to prepare the polymer solutions has a substantial effect on the morphology and spinnability of the fibres [6]. The boiling point, solubility of the polymer and dielectric constant of the solvent are all factors affecting the choice of solvent used in electrospinning.

During the electrospinning process, the solvent will evaporate as the fluid jet accelerates towards the collector [39]. Individual fibres will be formed if most of the solvent evaporates. While a fibre may not be formed at all, or a thin film of polymer solution may be deposited on the collector if most of the solvent does not evaporate [40]. The majority of polymers used in electrospinning are insoluble in aqueous solutions. Hence, the use of organic solvents is mostly unavoidable to allow for full polymer expansion in the solution, produce dry unmerged fibres, and make electrospinning feasible.

Most common solvents such as acrylic acid, chloroform, dichloromethane (DCM), tetrahydrofuran (THF), dimethylformamide (DMF), and hexafluoro isopropanol (HFIP) are used to synthesise electrospun fibres [41]. Table 2 shows a list of the common solvents used in the electrospinning process, together with the average fibre sizes of the generated nanofibres.

Even though these solvents have low boiling points and can dissolve most natural and synthetic polymers, it is important to note that some polymers have a high affinity for certain solvents and thus have a high likelihood of containing residual solvents during the electrospinning process. The residual solvent in electrospun fibres may be a potential cause of cytotoxicity, which can influence the biological function of a scaffold [42]. Therefore, it is very important to choose a compatible and suitable solvent before starting the electrospinning process.

**Table 2:** List of common solvents used in electrospinning based direct current (DC) and alternating current (AC) voltage.

Polymer	Solvent	Voltage	Fibre Diameter (nm)	Application	Reference
Silk fibroin/PCL/polyglycerol sebacate	1,1,1,3,3,3-Hexafluoro-2-propanol, formic acid	DC	4100 ± 3000–2110 ± 1340	Skin TE	[34]
PCL/HA/gelatin	Chloroform, methanol	DC	615 ± 269	Bone TE	[32]
Chitosan	Trifluoroacetic acid, dichloromethane	DC	231.2 ± 93.3	Bone regeneration	[35]
Collagen/polypyrrole/chitosan	Water/ethanol mixture	DC	337.9–83.7	Cardiac TE	[43]
Polyethylene glycol/acetate butyrate	Acetone, dimethylacetamide	DC	436.81 ± 139.52	Scaffold TE	[44]
PCL	Acetic acid/formic acid/acetone	AC	960 ± 400–2100 ± 1900	Industrial scale fabrication	[45]
Polyurethane/polyamide 6 (PA6)	-	AC	-	Suture	[46]
Fish skin gelatin (FG)/PCL	Glacial acetic acid	AC	237–313	Biomaterial	[47]
PCL	Formic acid, formic acid/acetic acid, and formic acid/acetic acid/acetone	AC	-	Industrial scale fabrication	[48]

## 5. Mechanism of Electrospinning

**Figure 3:**
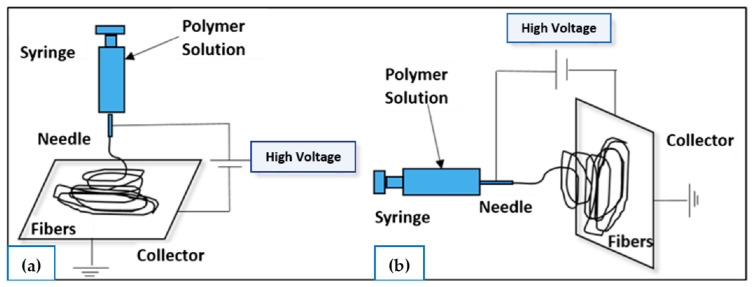
Schematic representation of the electrospinning process setup: (**a**) vertical setup and (**b**) horizontal setup of the electrospinning apparatus. Source: [50].

Electrospinning is a technique that involves a unique rotation process where it uses electrostatic force to produce nanofibres from a polymer solution. To overcome surface tension on a charged polymer solution, several tens of DC voltage are required to provide a significant repulsive electrical force [49]. As shown in Figure 3, there are currently two basic electrospinning setups: vertical and horizontal. Along with the rapid development of this technology, a more advanced system was created by a few research groups that can synthesise more complex nanofibrous structures in a more controlled and efficient manner [6]. In general, this process is conducted at room temperature under atmospheric conditions.

The syringe pump, grounded collector, and high-voltage DC power source are the three main components of the electrospinning setup. Prior to the process, the polymer must be dissolved with a solvent agent, such as chloroform, water, or ethanol, to form a polymer solution. In the electrospinning process, the syringe pump is used to force the solution through a needle attached to the syringe with a controlled flow rate. When the high voltage is applied to the solution, it induces a charge in the solution. As a result, it will cause a repulsive interaction between the like charges in the solution that increases with an increase in the electric field induced by the high voltage. A Taylor cone is formed when the electrical forces in the solution are balanced by the surface tension. When the electrical forces become greater than the surface tension of the solution, a charged fibre jet is ejected from the Taylor cone and accelerates towards a grounded collector [27].

Furthermore, there are some electrospinning strategies, such as the use of AC high-voltage electrospinning technology [45,46,47,48]. When the applied AC voltage is used, the electric pressure tends to suppress the capillary pressure because the polarity and magnitude of AC change with respect to time. At the critical voltage (positive and negative polarity), the jet is then ejected from the free liquid surface, and due to insufficient electric pressure, only a few of the previously formed jets are quenched at voltages below the critical voltage [48].

Another electrospinning strategy is the development of electrospinning-based scaffolds with a radially oriented nanofibre pattern. In order to collect the electrospun nanofibres with radially oriented structures, pin-ring-structured collectors were created by inserting a metal pin into the centre of a metal ring [51].

## 6. Electrospinning Parameters

Various parameters can significantly influence the smoothness and effectiveness of the electrospinning process, which can be divided into these categories: solution parameters, process parameters, and ambient parameters. Solution parameters include the viscosity, conductivity, molecular weight, and surface tension of the solution. While the process parameters include the applied voltages, distance from the tip to the collector, flow rate, and the electric field induced by the collector. Lastly, ambient parameters include the temperature and humidity of the surroundings. Each of these parameters affects the morphology and diameter of the nanofibre produced via the electrospinning process. Table 3 shows the effect of the morphology and diameter of the nanofibre produced when different parameters are applied during the electrospinning process.

## 7. Electrospun Nanofibres for TE Application

**Figure 4:**
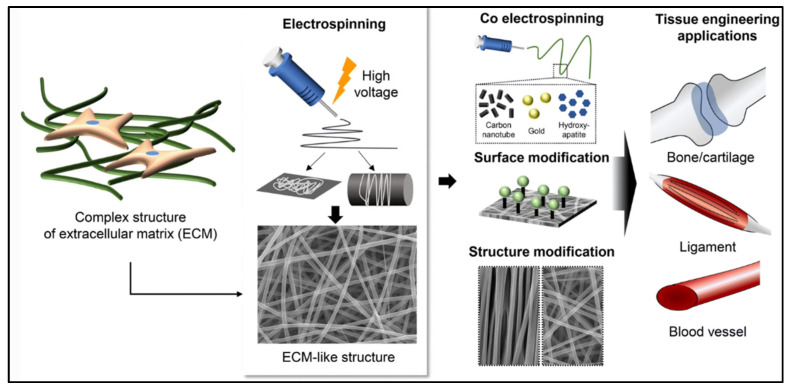
Application of scaffolds in TE. Source: [64].

Electrospun fibrous membranes are widely used for TE applications. TE requires a scaffold that supports cells, regenerates ECM components, and provides a vector to deliver biochemical factors [18]. The nanoscale structure of the native ECM-containing network of glycosaminoglycans (GAGs) and proteins forms a boundary between tissues and a supportive meshwork around the cells to provide cell anchorage [18]. Hence, many researchers have focused on designing scaffolds with similar properties to those of human tissue at the nanoscale level. Various scaffolds have been successfully produced from fibres through an electrospinning process which can be applied for bone, cartilage, vascular, nerve, and skin TE as shown in Figure 4.

The scaffold design used for TE should have good mechanical strength to support the new tissue growth and be bioactive to influence the cellular response. In addition, when deciding the usage of the fabricated material in clinical applications, a blood compatibility test must be considered as well. This is because blood incompatibility causes the formation of the thrombus during the platelet surface interaction, which results in the failure of the fabricated material [63]. Table 4 presents a summary of the previous research conducted on the application of scaffolds in TE.

### 7.1. Bone TE

Bone is an essential supportive structure in the human body. It serves to produce blood cells, enables human mobility, acts as a mineral pool for calcium and maintains the acid–base balance, and protects and supports various organs [81]. Natural bone is made up of 70 wt% hydroxyapatite nanocrystals and 30 wt% collagen fibrils [81]. Bone defects may cause decreased mobility, chronic pain, depression, sleep loss, and limitation in routine activities [81]. Bone healing become one of the main challenges to dentistry, orthopaedics, and craniofacial surgery. It is a difficult process that involves a cascade of osteogenic events [81]. Therefore, bone TE demands the production of engineered tissues that use scaffolds, cells, and growth factors to promote the regeneration of damaged or fractured bones with efficient mineralisation. The ideal bone healing scaffolds should have appropriate osteogenic differentiation ability and suitable mechanical properties [81].

Bone TE can have the following feature in biomimetic scaffolds: rebuilding of the nanofibrous collagen in ECM, high porosity to enable cell differentiation and ingrowth, and resistance to mechanical stress during tissue neogenesis [32]. Many scaffolds for bone TE have recently been developed using synthetic and natural polymers such as alginate, polylactic acid (PLA), poly(lactic-co-glycolic acid) (PLGA), chitosan, PCL, collagen, and their mixtures with other substances, producing fibrous nanocomposites with compositions and construction akin to the basic building blocks of inherently mineralised collagen nanofibres [64,82,83].

Gautam et al. prepared a nanocomposite scaffold which consists of gelatin, polycaprolactone, and nanohydroxyapatite for bone regeneration. The cell-scaffold constructs illustrated adequately spread cells and efficient cellular attachment [32]. Jaganathan et al. used the electrospinning technique to synthesise polyurethane electrospun nanofibres for bone repair. The addition of rosemary and copper sulphate to PU electrospun nanofibres provided better cell proliferation and higher tensile strength than the use of pure PU nanofibres [63]. Rethinam et al. developed an electrospun nano-bio membrane (ENBM) using PVA and a nano-demineralised bone matrix (nano-DBM) with the addition of carbon nanoparticles (CNPs) to give the ENBM extra strength for bone tissue regeneration [65]. The ENBM’s effective biocompatibility properties were investigated using the MG 63 osteoblast cell line, which demonstrated 100% biocompatibility and a comparatively significant number of viable cells. Furthermore, the ENBM scaffold exhibited improved mechanical properties, viz. 14.58 ± 0.13 MPa of tensile strength, 13.87% ± 0.05% of elongation at break, and 36.84% ± 0.11% water absorption [65]. This study showed the ENBM’s bone production in bone tissue regeneration and regenerative medicine.

### 7.2. Cartilage TE

Cartilage is a smooth, elastic, and resilient tissue. It consists of a rubber-like cushioning that covers and protects the ends of long bones at joints, as well as a structural component of the bronchial tubes, intervertebral discs, rib cage, ear, and nose. Treating a cartilage defect is a challenge for orthopaedics because of low mitotic activity, insufficient number of chondrocytes, and its avascular nature [81]. As a result, TE by electrospinning offers a viable alternative material for the treatment of injured cartilage. This is due to the ability of electrospun nanofibres made from synthetic, natural, or composite polymers to imitate the arrangement of collagen fibrils in the cartilage’s original ECM. Electrospun nanofibres can support the biological properties of scaffolds, such as chondrogenic differentiation and cell–matrix interaction, as well as promote the stiffness of the matrix [64].

Many studies have examined the potential of scaffolds with the use of nanofibres for cartilage healing. Sharifi et al. developed the electrospun composite nanofibrous scaffold, which consists of gelatin-chondroitin sulphate (G-CS) and PCL with a 2/1 ratio via the co-electrospinning technique for cartilage repair [66]. The presence of G-CS provided the scaffold with improved hydrophilicity and prepared the cell attraction environment for human mesenchymal stem cell (hMSC) cultures. Moreover, Feng et al. prepared a cartilage-derived extracellular matrix (cECM) and PCL to fabricate the cECM/PCL (mass ratio 50:50) hybrid nanofibres for cartilage regeneration. The presence of cECM in the nanofibrous membranes facilitated cartilage regeneration in vivo and promoted chondrocyte proliferation in vitro [67]. Silva et al. used the electrospinning technique to synthesise a scaffold consisting of PGS and PCL for cartilage repair. The composite scaffold showed a favourable result, as all the electrospun scaffolds prepared were able to mimic the structural features of cartilage ECM, which encourages the delivery of a chondro-inductive small molecule and supported cell culture. The electrospun scaffolds were nanoscale in size, which has previously been proven to be beneficial for mesenchymal stem cell (MSC) chondrogenic development. Hence, this finding highlights the prospect of kartogenin-loaded coaxial aligned nanofibres in the advancement of novel biomimetic MSC-based strategies to regenerate the articular cartilage, particularly for the repair of defects in its superficial zone [68]. Lastly, another biomedical application that can be used to treat the injured cartilage is Poly(N-isopropylacrylamide) based thermoresponsive surfaces. It involves the use of thermoresponsive cell culture dishes to fabricate the chondrocyte sheets. Cartilage regeneration was observed following the transplantation of chondrocyte sheets. Cartridge regeneration therapy is currently performed in human patients in clinical practice [84].

### 7.3. Vascular TE

One of the major causes of mortality and morbidity in the world is cardiovascular diseases such as coronary arterial restenosis, cardiomyopathy, and atherosclerosis. At the moment, autologous vascular grafts that are mainly used in clinics are limited by the need for a second surgical procedure and limited sources. Artificial grafts such as Dacron 1 and polytetrafluoroethylene have also been applied in the clinical treatment of vascular disease. However, these artificial grafts are not ideal for the treatment of small-diameter blood vessel disease (<5 mm) [81]. Electrospinning technology provides precise control over nanofibre orientation to modify the structure, porosity, and pore size distribution of the scaffolds. Therefore, electrospun scaffolds have shown promise in meeting the replacement needs of small-diameter blood vessels, as the electrospun nanofibres can mimic the structure of natural blood vessels [64].

Abdal-hay et al. studied the use of polyurethane, PCL, and tetrahydrofuran (THF) in the synthesis of biophasic scaffolds via the electrospinning technique [70]. The tensile strength and tensile elastic (Young’s) modulus of the biphasic scaffolds were significantly enhanced from 4.5 ± 1.72 and 45 ± 15 MPa (PU-only) to 67.5 ± 2.4 and 1039 ± 81.8 MPa (PCL/PU; *p* < 0.05) [70]. Additionally, the burst pressure, suture retention force, and compliance were all enhanced. Hence, this study could be used for vascular TE as it can improve the mechanical properties of a vascular graft scaffold [70]. Joy et al. prepared the oxidised carboxymethyl cellulose (OCMC)-gelatin tubular scaffold by using an electrospinning process. Its nontoxicity was validated by an MTT assay. Between 7 and 15 days after implantation, no aberrant foreign body reactions were noticed, suggesting that this tubular scaffold was appropriate for use in vascular TE applications [69]. Xiang et al. investigated the potential of spider silk protein (pNSR32) and gelatin (G) to incorporate into a PCL scaffold via the electrospinning technique [71]. The results showed that the pNSR32/PCL/Gt scaffold had a faster degradation rate, wider fibre diameter distribution, and larger average fibre diameters than the pNSR32/PCL and PCL scaffolds. The pNSR32/PCL/Gt scaffold had greater tissue and blood compatibility than the PCL and pNSR32/PCL scaffolds, which was supported by this study. The pNSR32/PCL/Gt scaffold was a suitable option for creating small-diameter vascular tissue due to the absence of an inflammatory factor and the induction of genotoxicity releases [71].

Klabukov et al. explored flat microfibrous scaffolds obtained by electrospinning polycaprolactone with the incorporation of the pCMV-VEGF-165 plasmid into the microfibres at concentrations of 0.005 ng of plasmid per 1 mg of polycaprolactone (0.005 ng/mg) (LCGroup) and 0.05 ng/mg (HCGroup) [85]. The results demonstrated that pCMV-VEGF165 plasmid functionalisation of polycaprolactone led to better vascularisation 33 days after implantation; however, vessel growth did not appear to be correlated with scaffold degradation rate [85].

### 7.4. Nerve TE

A common burden on the global healthcare system is neurological deterioration. Although artificial nerve grafts and the implantation of autografts have been investigated for the treatment of nerve damage, nerve damage restoration is still a difficult issue in this sector. This is due to the fact that autografting lacks accessible donors, requires surgery at the donor location, and may cause the donor site to lose its functionality, while synthetic nerve grafts perform poorly at repairing nerve abnormalities [64]. Therefore, an alternate strategy for the treatment of nerve injury could involve the use of scaffolds made from electrospun nanofibres. Improved neurite outgrowth, the possibility to build nanofibre-based artificial nerve grafts, and mechanical and biomechanical cues for differentiating stem cells are all advantages of electrospun nanofibres [64]. Applications of aligned nanofibrous scaffolds or the incorporation of bioactive materials into electrospun nanofibrous scaffolds have been the focus of some studies [81].

An electrospun PCL-gelatin-graphene nanofibrous mat was designed by Heidari et al., which exhibited 99% antibacterial properties against gram-positive and gram-negative bacteria [75]. These superior properties, along with an enhancement in the biodegradation features and hydrophilicity, have made the PCL/gelatin/graphene nanofibre a favourable candidate to be used as electrically conductive scaffolds in nerve TE [75]. In addition, Ghasemi-Mobarakeh et al. used the electrospinning technique to fabricate a biocomposite of the PCL/gelatin scaffold [76]. The MTS assay and SEM results showed that the biocomposite of the PCL/gelatin 70:30 nanofibrous scaffolds enhanced nerve differentiation and proliferation compared to PCL nanofibrous scaffolds and acted as a positive cue to support neurite outgrowth. It was found to exhibit the most balanced properties to meet all the required specifications for nerve tissue [76]. Babaie et al. prepared a PVA and poly (3,4-ethylenedioxythiophene) (PEDOT) scaffold via electrospinning for nerve treatment. There was an improvement in terms of cell viability and physiochemical properties when a PVA/PEDOT-containing scaffold was used [77]. This study showed that the PVA/PEDOT scaffold could enhance neural differentiation and cellular response by mimicking the properties of the native neural tissue [77].

### 7.5. Skin TE

Skin is the first defence organ against insult from the environment and is the largest organ of vertebrates. It protects vertebrates from chemical hazards, mechanical injury, and bacterial invasion. The body triggers a response called wound healing when an injury damages the skin’s protective barriers. Usually, skin necrosis and defects can result from chemical damage, tissue trauma, burns, and ultraviolet radiation. Hence, skin defect repair is an important clinical issue. Allografts, autografts, and xenografts are the traditional methods for repairing skin defects [64]. However, these conventional treatments have their own disadvantages, as autografts are still limited by donor site morbidity and the autograft supply. Although it is easy to obtain xenografts and allografts, they are constrained by a significant risk of disease transmission and immunological rejection. That is why a lot of research has been conducted on electrospun scaffolds for the treatment of skin defect repair [86,87]. This is because they can easily load epidermal factors, vasculogenic factors, angiogenic factors, and molecules with antimicrobial and anti-inflammatory properties. They can also spread and aid in the adhesion of keratinocytes and fibroblasts. Additionally, they can aid mesenchymal stem cells in differentiating and expanding to form epidermal lineage cells [64].

Many synthetic and natural polymers have been developed as matrix biomaterials for wound healing. Adeli-Sardou et al. investigated the potential of lawsone (2-hydroxy-1,4-naphthoquinone) and polycaprolactone-gelatin (PCL-G) to fabricate a scaffold via the electrospinning technique for wound healing [79]. The PCL/G/Law 1% scaffold increased cell attachment and proliferation significantly. It also had the highest impact on healing by increasing the re-epithelialisation of the wound after 14 days. Thus, it was concluded that PCL/G/Law 1% scaffold has excellent characteristics and can be used for skin tissue regeneration [79]. Narayanan et al. fabricated a scaffold via the electrospinning technique, which consisted of glucose-reduced graphene oxide (GRGO), PVA, and glutaraldehyde (GA). The findings revealed that after 21 days of culture, the scaffold considerably improved metabolic activity and had great compatibility with fibroblasts. The scaffold improved fibroblast vitality and proliferation, according to live/dead imaging studies, suggesting the possibility of skin TE applications [78]. Keirouz et al. prepared a scaffold of PGS and polyvinyl pyrrolidone (PVP) by using an electrospinning technique. Good viability and proliferation of human dermal fibroblast cells were shown in an in vitro examination of the PGS/PVP scaffold [80]. This study introduced a special elastomer as a possible future contender for skin substitute structures, and it gave useful insights for adjusting the elastic characteristics of an electrospun material [80].

### 7.6. Tendon and Ligament TE

Due to their innately poor healing potential for tendon and ligament injuries—which are typically common in physically active young people—they present a substantial clinical problem. Natural healing of tendon and ligament injuries usually forms scar-like tissue that has poor mechanical properties due to their innately poor healing potential. So, a possible alternative strategy for tendon and ligament regeneration is tissue engineering. By providing an artificial ECM that is analogous to the collagen fibre bundles of the real tissues, electrospun nanofibrous scaffolds have been used to aid in the regeneration of ligaments and tendons [64]. Collagen fibres arranged in parallel arrays and tightly packed together make up the majority of healthy normal tendons, giving them highly anisotropic mechanical properties [64]. Because they resemble the anisotropic shape of the natural tissues, scaffolds developed of aligned nanofibres are, therefore, potential options for tendon and ligament tissue engineering.

Jiangyu Cai et al. created novel nano-micro fibrous woven scaffolds with tendon-like anisotropic structures and high-strength mechanical properties for the treatment of large-size tendon injury by combining an advanced electrospun nanofibre yarn-generating technique with a conventional textile manufacturing strategy [88]. The mechanical qualities of the electrospun nanofibre yarns, which were created from pure poly L-lactic acid (PLLA) or a combination of silk fibroin (SF) and PLLA, were on par with or even better than those of commercial PLLA microfibre yarns [88].

## 8. Clinical Perspectives of Electrospun Nanofibres

Although electrospinning is a cheap, simple, and versatile method to prepare fibrous scaffolds by using a nanometre which is suitable for TE, its clinical application has not yet been fully exploited in the market. Several companies have made significant technical progress in this field, but none of the products have yet been approved by the Food and Drug Administration (FDA) [89]. For example, St. Teresa Medical, Inc.^®^ (Eagan, MI, USA) produced SURGICLOT, in which electrospun fibres provide proteins to promote blood clotting [90]. Another example is as follows: Zeus^®^ (Orangeburg, SC, USA) produced an electrospun PTFE graft named Bioweb™ (Orangeburg, SC, USA) with application in stent encapsulation, scaffolding, and implantable structures in the body [91]. Furthermore, Nicast synthesised a vascular access graft, AVflo™ (Chennai, India), prepared from silicone and polycarbonate-urethane with a multilayered electrospun configuration [64].

The above scenario could be related to problems with the effectiveness and safety of electrospun fibres as well as the financial and technological obstacles that need to be overcome for clinical applications. Additionally, electrospinning has a low productivity yield from an economic perspective and needs highly skilled workers to produce items of a high calibre. Technically speaking, one of the key problems is the dearth of reliable and sophisticated fabrication techniques, as well as the subpar product quality. For instance, it is still very difficult to produce a large number of commercial goods using an electrospinning setup in a continuous process. By overcoming the abovementioned difficulties, the immense promise of electrospun nanofibres in TE may be exploited and converted into clinical results [64].

## 9. Limitation of Electrospun Scaffold

Although the electrospinning technique has a clear advantage over other scaffold fabrication techniques, this technique still encounters two main practical limitations, namely poor cell infiltration and inadequate mechanical strength for load-bearing applications [40].

### 9.1. Poor Cellular Infiltration and Ingrowth

Electrospun scaffolds have a high surface porous structure and are made entirely of layers of densely packed nanofibres. Electrospun materials’ porous structure enables the passage of nutrients and growth agents through the mesh. In general, the fabrication of electrospun fibres on the submicron scale increases the number of fibre contacts per unit length, which results in a decrease in the average pore radius. Electrospun nanofibres with extremely small pore sizes can lead to poor cellular infiltration. The high packing density of the nanofibrous mat is also a factor in the poor cell infiltration in the electrospun nanofibrous scaffold. The grounded charge on a conventional electrospinning collector is uniformly distributed over a considerable region. As a result, a group of fibres are deposited next to one another in one layer, and then other layers are deposited on top of the first one. The layers below are compressed because each layer is still securely linked to the grounded collector. The result of this procedure is a structure with tightly packed fibre layers [40]. Poor cellular infiltration may block cell migration into the scaffold, limiting vascularisation, resulting in an uneven distribution of cells throughout the structure, and preventing tissue ingrowth due to the limited transport of nutrients and waste [6]. Therefore, scaffold design should include interconnected and larger pores for better cellular and tissue interaction.

### 9.2. Inadequate Mechanical Strength for Load Bearing

Although electrospun scaffolds can mimic the nanomechanical and nanostructural features of the native ECM, these scaffolds lack the appropriate mechanical properties for load-bearing applications because the scaffolds inherently possess high porosity. Furthermore, the electrospun scaffolds also cannot be utilised for load-bearing applications as mechanical strength of approximately 150 MPa is required, which is not attainable with conventional electrospun fibres [40]. Therefore, a more robust technique is required to overcome this limitation, as discussed in the next section.

As the mechanical properties of scaffold materials are essential for successful tissue engineering applications, it is critical to fabricate electrospun scaffolds with adequate mechanical properties, especially for load-bearing applications. The scaffolds must be sufficiently strong to support cell spreading, cell adhesion, and ECM synthesis. In addition, they should be able to withstand external forces acting upon them to support their function without collapsing and maintain their integrity.

## 10. Solution for Overcoming the Electrospun Scaffold Limitation

Several solutions have been attempted to overcome poor cellular infiltration, such as tuning electrospinning parameters, electrospinning using a sacrificial component, and a combination of nano- and microfibres.

While with respect to inadequate mechanical strength, the incorporation of the inorganic phase with electrospun materials and postprocessing modification are the best methods to overcome this limitation, as doing so can fabricate electrospun scaffolds with increased structural and mechanical strength [40]. Furthermore, several experiments have been conducted to produce multilayered and heterogeneous scaffolds with predictable mechanical properties [92,93].

### 10.1. Tuning Electrospinning Parameters

During the electrospinning process, electrospinning parameters such as flow rate, applied voltage, distance from the tip to the collector, concentration of polymer, and ambient conditions considerably affect the structure and morphology of nanofibres. Adjusting these electrospinning parameters is one of the simplest ways to obtain the desirable structure of nanofibres, as by doing so, we can directly tune the pore dimension and packing density of the nanofibres. For example, increasing the flow rate, polymer concentration, or solution viscosity will increase the nanofibre diameter, which will lead to larger pore space [40]. Previous studies on different methods to tune the electrospinning parameters used to overcome poor cell invasion in the electrospinning scaffolds described in this review are summarised in Table 5.

### 10.2. Electrospinning Using a Sacrificial Component

Various materials such as salt particles, water-soluble polymers, and ice crystals can be temporarily combined with shaped nanofibres to act as removable templates during electrospinning. After the removal of the template, more porosity is introduced into the network due to the voids that arise between the electrospun fibres. Rapidly dissolving polymers such as PVA, gelatin, or polyethylene oxide (PEO) are commonly fused into slowly degradable polymers by dual electrospinning. After soaking the nanofibres in an aqueous medium, nanofibres with relatively large pore sizes and improved porosity are obtained. The pore size of these materials depends on the shape of the sacrificial fibre phase entangled in the electrospun structure [40]. Moreover, the role of sacrificial fibres is not limited to facilitating cell invasion but also to creating loosely connected structures formed by selective lysis.

Recently, salt leaching has been widely used to fabricate porous 3D scaffolds for TE applications. The salt particles are uniformly dispersed in the polymer solution and leached out to create large pores with controllable pore sizes determined by the particle size. Therefore, the combination of electrospinning and salt leaching results in electrospun scaffolds with larger diameters and pore sizes for improved cell invasion [97]. Kim created his macroporous and nanofibre 3D scaffolds by saline leaching [98]. In their study, a homogenous porous mesh of hyaluronic acid/collagen was created by rotating salt particles simultaneously. Sodium chloride (NaCl) particles with deposited nanofibres water-swellable frameworks with macroporous and nanofibre morphologies were obtained by chemical crosslinking and subsequent salt leaching. The resulting scaffolds promoted chondrocyte proliferation and growth and maintained their morphology during the culture period [98]. In another study by Nam et al., small salts, approximately 90–106 μm in diameter, were used in Taylor cones with a sheath surrounding the needle at intervals of electrospun polycalolactone. This created a uniform nanofibre network with well-dispersed salt particles. Salt leaching then increased delamination within the PCL fibre framework, resulting in larger and improved pores [99].

### 10.3. Combination of Micro- and Nanofibres

Increasing the fibre diameter improves cell infiltration and pore size but decreases the fibre-cell contact area. An ideal tissue engineering scaffold should promote both good cell invasion and good adhesion, ultimately requiring a balanced combination of both to promote complete tissue formation. Therefore, it is important to find a balance between pore size and fibre diameter to ensure good cell infiltration. A combination of microfibres and nanofibres offers a solution for optimised cell invasion and cell adhesion with critical pore connectivity. Nanofibres provide an ECM-like substrate for enhanced cell attachment and proliferation, while microfibres can enlarge the pore size and promote proper cell invasion [40]. The pore size was shown to strongly depend on the nanofibre diameter, ultimately determining the cell permeation behaviour of the electrospun scaffolds [96]. This leads to a method of combining nanofibres and microfibres to create scaffolds that exploit the inherent advantages of both electrospun fibres. Electrospun nano/microfibre hybrid scaffolds can be generated by two-stream electrospinning, where one stream produces nanofibres and the other produces microfibres [97].

Ju et al. showed to overcome the limitation of smooth muscle cell infiltration into the scaffold by fabricating a two-layer electrospun scaffold composed of large-diameter fibres on the outside and small-diameter fibres on the inside. The resulting scaffolds exhibited endothelialisation at the luminal surface as well as SMC penetration into the outer layer [100]. In another study, Pham et al. produced a polycaprolactone (PCL) scaffold consisting of 5 mm microfibres interspersed with 600 nm nanofibres within a bioreactor within 12 days, supporting complete cell infiltration throughout the scaffold [101].

A study on the salt-based electrostatic flocking method by McCarthy et al. was used to fabricate a two-layer electrospun scaffold composed of poly(ε-caprolactone) (PCL) microfibres (MF) and electrospun PCL nanofibre yarns (NFY). Both MF and NFY were evaluated for mechanical properties, swarm functionality and biological responses. The resulting scaffolds have the potential for wound healing and treatment of lumbar degenerative disc disease [102].

### 10.4. Incorporating the Inorganic Phase with Electrospun Materials

Electrospinning with copolymers is widely pursued in TE applications through methods such as copolymerisation and incorporation of inorganic phases, as it offers improved properties of polymeric materials, such as mechanical strength, thermal stability tuning, and barrier properties [6]. It has been observed that mixing an inorganic material such as nanohydroxyapatite bioactive nanoparticles or carbon nanotubes into the polymer matrix alters physical properties and biodegradation, thereby resulting in a modified polymer with highly influenced mechanical properties. These characteristics are desired in TE compositions such as scaffolds, fibres, and films. When these components are present in scaffold materials, they can boost mechanical strength when compared to pure polymeric electrospun structures.

For example, hydroxyapatite (HA) is the major inorganic component of natural bone widely used for incorporation into the polymer matrix. Several approaches have been used for the incorporation of HA into an electrospun scaffold, such as blending of HA with a polymeric component, an alternate soaking method, incubating electrospun scaffolds in simulated body fluid (SBF) [38,103,104] and also the use of HA with emulsion and coaxial electrospinning to create multifunctional scaffolds for bone tissue regeneration [105].

### 10.5. Postprocessing Modification

Most electrospun scaffolds of natural polymers are not very stable in aqueous environments. They collapse and swell into films when they come into contact with water, as the number of interconnected pores is significantly reduced. Moreover, they show a remarkable reduction in tensile strength [40]. Thus, crosslinking is applied to enhance the mechanical properties and maintain the fibrous morphology after electrospinning [106]. Crosslinking can be performed either by radiation, including γ-irradiation or ultraviolet heat, dehydrothermal treatment, or chemical agents, including glutaraldehyde [107,108,109]. Therefore, crosslinking has been shown to be an effective method for enhancing the mechanical strength and the water resistance of fibrous scaffolds. For example, Jiang managed to enhance the mechanical properties and the water stability of the electrospun fibres of a plant protein, zein, through a two-step crosslinking process [110]. A pre-crosslinking step was carried out to allow the formation of crosslinking sites and the expansion of the zein molecule chains. After the electrospinning process, the electrospun zein materials were post-crosslinked by heating. The resultant scaffolds showed morphological stability and improved mechanical strength [110].

## 11. Conclusions

A nanofibrous scaffold for TE applications can be synthesised using one of the three main scaffold fabrication techniques, namely electrospinning, self-assembly, and phase separation. Of these techniques, electrospinning is the most studied and seems to provide the most promising results for TE. Electrospinning is a dry spinning process that uses electrostatic forces to draw fibres from a polymer solution. Electrospinning has been shown to be a powerful tool for fabricating TE scaffolds because it is inexpensive, versatile, simple, and can form ECM-like structures. Nanofibres synthesised using electrospinning technology have resulted in scaffolds with increased porosity and a large surface area. These properties have been shown to provide a favourable environment for cell adhesion, proliferation, and differentiation. Therefore, nanofibrous scaffolds are currently being explored as scaffolds for skin, heart, bone, nerves, and vessels TE. However, for final use in clinical applications, the poor cell infiltration and low mechanical strength of conventional electrospun scaffolds should be considered. These inherent limitations of electrospinning have recently attracted much attention, and various solutions have been proposed to overcome them. Despite these limitations, previous studies have clearly shown that nanofibre-based scaffolds have excellent potential for developing a wide range of applications TE. Future research should investigate multifunctional scaffolds capable of physically promoting cell development and supporting tissue regeneration by delivering bioactive signals to make them useful for the clinical applications market. It is critical to move the production of electrospun nanofibres from the laboratory to the commercial scale by enabling researchers from multiple disciplines to fabricate and design innovative tissue engineering substrates with the desired goals [111]. For the future prospect, with the right materials, optimised spinning parameters and carefully selected spinning medium, it is possible to develop the electrospinning technique to produce novel materials for a wide range of applications [112].

## Figures and Tables

**Table 1:** Benefits and drawbacks of several techniques for scaffold fabrication.

Scaffold Fabrication Method	Benefits	Drawbacks	References
Electrospinning	- Ability to control over the diameters and morphology of the micro- and nanoscale thin fibres. - Ease in production - Ability to generate homogeneous mixtures with nanoscale fibres.	- Sometimes, solvent used might be cytotoxic due to the use of a wide range of biomaterials. - Need a high-voltage apparatus to operate electrospinning.	[18,19]
Self-assembly	- Possess high porosities (80–90% porosity). - Provide high cell viability (70–90%).	- Process is complex and time-consuming. - Poor control over fibre dimension.	[18,20]
Phase separation	- Ease in production. - Possess high porosities (60–95% porosity).	- Poor control over architecture. - Restricted range of pore sizes. - Use of organic solvent.	[18,19]

**Table 3:** Electrospinning parameters (solution, processing, and ambient) and their effects on fibre morphology.

Parameter	Effects on Morphology of Nanofibre	References
**(1) Solution parameters**		
Viscosity	Less number of beads produced; increase in fibre diameter as viscosity increases.	[52,53]
Polymer concentration	The fibre diameter increases as the polymer concentration increases.	[54,55,56]
Molecular weight of polymer	The number of beads and droplets decreases with an increase in the molecular weight of the polymer.	[57]
Conductivity of polymer	Higher conductivity of the polymer causes the fibre diameter to decrease.	[52,57]
Surface tension	There is no change in fibre morphology; high surface tension leads to instability of the jet.	[58]
**(2) Process parameters**		
Applied voltage	The fibre diameter decreases as the applied voltage increases.	[56,59]
Distance from tip to collector	Large distance from the tip to the collector leads to small production of beads; minimum distance is required to synthesise a uniform fibre.	[60,61]
Flow rate	The reduction in fibre diameter is proportional to the reduction in flow rate.	[24]
**(3) Ambient parameters**		
Humidity	High humidity forms pores on the surface of the fibres.	[62]
Temperature	An increase in temperature results in a smaller fibre diameter.	[62]

**Table 4:** Several applications of electropun scaffolds in TE.

Application	Polymer/Material	Solvent	Result	Reference
Bone TE	- Gelatin (G) - Polycaprolactone (PCL) - Nanohydroxyapatite (HA)	- Acetic acid Chloroform/methanol	- The nanocomposite scaffold, 20 min exhibited average fibre diameter of 615 ± 269 nm and average pore size 4.7 ± 1.04 μm. - Cell-scaffold constructs illustrated adequately spread cells and efficient cellular attachment.	[32]
	- Polyurethane (PU) - Rosemary (RM) oil - Copper sulphate (CuSO_4_)	- Dimethylformamide (DMF)	- The electrospun pure PU nanofibres had an average diameter of 875 ± 154 nm, while as-spun PU/RM and PU/RM/CuSO_4_ nanofibres exhibited diameter of 745 ± 133 nm and 414 ± 156 nm, respectively. - Addition of RM and CuSO_4_ to PU electrospun nanofibres provides better cell proliferation and gives higher tensile strength than those of the pure PU nanofibres.	[63]
	- Nano-demineralised bone matrix (nano-DBM) - Polyvinyl alcohol (PVA) - Carbon nanoparticles (CNP)	- Acetone - Chloroform	- Addition of CNP gives additional strength to the electrospun nano-bio membrane (ENBM). ENBM scaffold prepared using PVA, nano-DBM, and CNP (0.6%) exhibits improved mechanical properties, viz. 14.58 ± 0.13 MPa of tensile strength, 13.87% ± 0.05% of elongation at break, and 36.84% ± 0.11% water absorption. - The effective biocompatibility properties of ENBM were evaluated using MG 63 osteoblast cell line, which showed 100% biocompatibility and more viable cells present in the electrospun ENBM.	[65]
Cartilage TE	- Chondroitin sulphate (CS) - Gelatin (G) - Polycaprolactone (PCL).	- Acetic acid/formic acid - 2,2,2-Trifluoroethanol/water	- Electrospun composite nanofibrous scaffold fabricated by co-electrospinning technique of G-CS and PCL with 2/1 ratio provides a scaffold with improved hydrophilicity and prepares cell attraction environment for cultured human mesenchymal stem cells (hMSCs).	[66]
	- Cartilage-derived extracellular matrix (cECM) - Polycaprolactone (PCL)	- Hexafluoro-2-propanol (HFIP)	- The cECM/PCL (mass ratio 50:50) hybrid nanofibres appeared to be thinner, smoother, and more uniform with enhanced mechanical properties and wettability than the electrospun PCL. - The presence of cECM in the nanofibrous membranes facilitated cartilage regeneration in vivo and promoted chondrocyte proliferation in vitro.	[67]
	- Polyglycerol sebacate (PGS) - Polycaprolactone (PCL)	- 2,2,2-Trifluoroethanol	- All the electrospun scaffolds produced were able to mimic the structural features of cartilage ECM, which promoted the delivery of a chondro-inductive small molecule and supported cell culture. The size of the electrospun scaffolds was in the nanometer scale, which was previously shown to be advantageous for mesenchymal stem (MSC) chondrogenic differentiation. - This finding highlighted the potential of kartogenin-loaded coaxial aligned nanofibres for the advancement of novel biomimetic MSC-based strategies to regenerate articular cartilage, particularly for the repair of defects in its superficial zone.	[68]
Vascular TE	- Oxidised carboxymethyl cellulose (OCMC) - Gelatin (G)	- Acetic acid	- MTT assay confirmed its no-toxicity, and no abnormal foreign body reaction was observed on day 7 and day 15 after implantation, which made this tubular scaffold suitable for use in vascular TE applications.	[69]
	- Polyurethane - Polycaprolactone (PCL)	- Tetrahydrofuran (THF)	- This research aims to improve the mechanical properties of a vascular graft scaffold. - The tensile strength and tensile elastic (Young’s) modulus of the biphasic scaffolds were significantly enhanced from 4.5 ± 1.72 and 45 ± 15 MPa (PU-only) to 67.5 ± 2.4 and 1039 ± 81.8 MPa (PCL/PU; *p* < 0.05). Additionally, burst pressure, suture retention force, and compliance were all enhanced. - Hence, this study could be potentially extended to that of other biphasic scaffolds to better improve the mechanical properties of the electrospun fibre mat.	[70]
	- Gelatin (G) - Spider silk protein (pNSR32) - Polycaprolactone (PCL)	- Formic acid	- G and pNSR32 were used in this study to improve the cytocompatibility of the electrospun pNSR32/PCL/Gt scaffold. - The result showed that the pNSR32/PCL/G scaffold had faster degradation rate, wider fibre diameter distribution, and larger average fibre diameters than the pNSR32/PCL and PCL scaffolds. - In conclusion, this study validated that the pNSR32/PCL/G scaffold exhibited better tissue and blood compatibility than the PCL and pNSR32/PCL scaffolds. In addition, there was no inflammatory factor, and the induction of genotoxicity releases made the pNSR32/PCL/G scaffold a good candidate for engineering small-diameter vascular tissue.	[71]
Cardiac TE	- Polycaprolactone (PCL) - Polyglycerol sebacate (PGS)	- Acetic acid	- PCL/PGS blends and neat PCL showed defect-free microstructures, whereby the average fibre diameter increased with the addition of PGS (0.8 ± 0.3 µm and 1.3 ± 0.7 µm, respectively) - PCL/PGS fibres fabricated with acetic acid proved to be potentially suitable for application in cardiac TE as their mechanical properties and biodegradability were better than those of fibres made using conventional solvents. This was attributed to the fact that the acetic acid would fully evaporate during electrospinning, leaving no toxic residues or hazards behind.	[72]
	- Polyglycerol sebacate (PGS) - Corn protein zein	- Acetic acid	- In vitro degradation studies in PBS revealed a physicochemically stable system over a 28-day period, showing only a slight drop in pH after 28 days. - The results indicated that the novel PGS/zein fibrous structures could be valuable for cardiac patch applications as these fibres took advantage of the biocompatibility of zein to fabricate microfibres with the improved mechanical stability and attractive mechanical properties of PGS.	[73]
	- Solubilised cardiac extracellular matrix (ECM) - Alginate - Chitosan	- Deionised water - Hydrochloric acid	- The porosity of the scaffolds was more than 96% with a very high swelling rate, while their stability was maintained in a PBS solution. - Mixing ECM with alginate and chitosan drastically improved the tensile strength of ECM. Moreover, mixing ECM with polysaccharides in the ratio of 75:25 (E75/P25) improved the proliferation of the scaffolds. - Scanning electron microscopy (SEM) revealed the presence of hMSCs cells and the porous structure inside the pores. Histological analysis confirmed that cardiomyocyte penetration inside the scaffolds after 7 days of culture.	[74]
Nerve TE	- Polycaprolactone (PCL) - Gelatin (G) - Graphene	- Acetic acid	- Electrospun PCL/G/graphene nanofibrous mats exhibited 99% antibacterial properties against gram-positive and gram-negative bacteria. - These superior properties, along with an enhancement in the biodegradation features and hydrophilicity, have made the PCL/G/graphene nanofibre a promising candidate for use as electrically conductive scaffold in nerve TE.	[75]
	- Polycaprolactone (PCL) - Gelatin (G)	- Hexafluoro-2-propanol	- MTS assay and SEM results showed that the biocomposite of the PCL/G 70:30 nanofibrous scaffolds enhanced nerve differentiation and proliferation compared with the PCL nanofibrous scaffolds and acted as a positive cue to support neurite outgrowth. - It was found to exhibit the most balanced properties to meet all the required specifications for nerve tissue and was used for an in vitro culture of nerve stem cells (C17.2 cells).	[76]
	- PVA - PEDOT (poly(3,4-ethylenedioxythiophene))	- Deionised water	- There was improvement in terms of cell viability and physiochemical properties when using PVA/PEDOT-containing scaffolds. - This study showed that a PVA/PEDOT scaffold could enhance neural differentiation and cellular response by mimicking the properties of the native neural tissue.	[77]
Skin TE	- Glucose-reduced graphene oxide (GRGO) - PVA - Glutaraldehyde (GA)	- Acetone	- Results showed that the scaffold exhibited excellent compatibility with fibroblasts and significantly increased the metabolic activity after culture for 21 days. - Live/dead imaging assays showed that the scaffold increased the fibroblast viability and proliferation, thus indicating the potential for skin TE applications.	[78]
	- Lawsone (2-hydroxy-1,4-naphthoquinone) - Polycaprolactone-gelatin (PCL-G)	- 2,2,2-Trifluoroethanol - Dimethylformamide (DMF)	- The PCL/G/Lawsone 1% scaffold increased cell attachment and proliferation significantly. It also had the highest impact on healing by increasing the re-epithelialisation of the wound after 14 days. - Thus, it was concluded that the PCL/G/Lawsone 1% scaffold had excellent characteristics and could be used for skin tissue regeneration.	[79]
	- Polyglycerol sebacate (PGS) - Polyvinyl pyrrolidone (PVP)	- Dimethylformamide (DMF) - 1,1,1,3,3,3-Hexafluoro-2-propanol (HFIP)	- In vitro examination of the PGS/PVP scaffold showed good viability and proliferation of human dermal fibroblast cells. - This research provided valuable insights for tuning the elastic properties of the electrospun material by incorporating this unique elastomer as a promising future candidate for skin substitute constructs.	[80]

**Table 5:** Different methods of tuning electrospinning parameters for overcoming the poor cellular infiltration in electrospun scaffolds.

Method Used	Polymer	Solvent	Result	Reference
Increasing polymer concentration	Polycaprolactone (PCL)	Dimethylformamide (DMF) and Dichloromethane (DCM)	- Increased in diameter and pore size of nanofibres. - Improved cell infiltration. - The best cellular infiltration was attained when the pore size was close to the size of the target cell.	[94]
Increasing solvent evaporation via heat localisation in the path of fluid jet	Poly(l-lactic acid) (PLLA)	Dichloromethane (DCM) and Dimethylformamide (DMF)	- Increased thickness, pore diameter, and porosity of the nanofibres. - High amount of cell infiltration along with cell distribution.	[95]
Increasing flow rate	Polycaprolactone (PCL)	Chloroform	- Increase in nanofibre diameter. - Enhanced cell infiltration.	[96]
	Elastin	Hexafluoroisopropanol	- Increase in diameter and thickness of nanofibres. - Decrease in nanofibre density. - Improved cell infiltration.	[97]

## Data Availability

The data are available upon request to the authors.
